# No evidence of intra-articular knee deformity following growth arrest through temporary epiphysiodesis – a retrospective study of 81 patients

**DOI:** 10.1186/s13018-025-05875-0

**Published:** 2025-05-17

**Authors:** Andrea Laufer, Jan Disselkamp, Georg Gosheger, Gregor Toporowski, Adrien Frommer, Veronika Weyer-Elberich, Robert Roedl, Bjoern Vogt

**Affiliations:** 1 Paediatric Orthopaedics, Deformity Reconstruction and Foot Surgery, Muenster University Hospital, Albert-Schweitzer-Campus 1, Muenster, 48149 Germany; 2 General Orthopaedics and Tumour Orthopaedics, Muenster University Hospital, Muenster, Germany; 3 Institute for Biometry and Clinical Research, Muenster University Hospital, Muenster, Germany

**Keywords:** Temporary epiphysiodesis, Growth modulation, Leg length discrepancy, Tall stature, Knee joint morphology, Intra-articular deformity, Volcano effect, Secondary coronal angular deformity, Mechanical axis deviation, Children and adolescents

## Abstract

**Background:**

Temporary epiphysiodesis (tED) around the knee is a well-established treatment approach for leg length discrepancies (LLD) in skeletally immature patients. Moreover, it may be conducted bilaterally to reduce height in tall stature. However, secondary changes in the bony morphology of the tibial plateau after tED have been reported. This study thus aimed to evaluate secondary alterations in knee joint morphology following tED around the knee.

**Methods:**

Radiographs of 81 skeletally immature patients aged 7-15 years were retrospectively analysed. 10/81 patients underwent bilateral tED with RigidTacks^TM^ (RT) to reduce growth in tall stature, whereas 71/81 patients (35 with eight-Plates^TM^ (EP), 36 with RT) received unilateral treatment for LLD. To assess changes in knee joint morphology, following radiographic parameters were evaluated: femoral floor angle (FFA), tibial roof angle (TRA), width at femoral physis (WFP), and femoral notch-intercondylar distance (FNID). Furthermore, mechanical axis deviation (MAD), mechanical lateral distal femoral angle, medial proximal tibial angle, and joint line convergence angle were measured to analyse coronal alignment. All parameters were assessed prior to implantation, prior to device removal, and at last follow-up.

**Results:**

Mean treatment duration was 2.7 years (standard deviation (SD) 1.1). Statistically relevant changes were observed in WFP (p=0.025), FNID (p=0.008), and MAD (p=0.002) after tED using EP, and in FNID (p=0.043) using RT. Compared with reference values for untreated healthy children, these relevant changes remained within one SD. Mean absolute MAD change using EP was 3.9 mm (SD 7.1) compared to 1.9 mm (SD 8.8) using RT. Secondary coronal malalignment with need for revision surgery was found in 11/81 patients (4/35 with EP, 7/46 with RT).

**Conclusions:**

Statistically relevant changes in bony morphology after tED were only observed in the distal femur. However, there was no evidence of intra-articular knee deformities as all measured femoral and tibial parameters remained within physiological margins and were considered clinically inconsequential. Nevertheless, there was a considerable number of patients with secondary coronal malalignment among both implant groups, necessitating further elucidation.

## Background

Unilateral growth arrest through epiphysiodesis of the growth plates adjacent to the knee joint is a well-established procedure for the treatment of leg length discrepancies (LLD) in skeletally immature patients [1, 2]. Furthermore, epiphysiodesis may be conducted bilaterally to reduce final height in tall stature [3, 4]. Even though implant-mediated temporary epiphysiodesis (tED) has shown satisfactory results for both indications [3–5], it has recently been associated with central knee deformities, in particular regarding secondary alterations of the bony morphology of the proximal tibia [5, 6]. This is of particular note since, for leg length equalisation, as well as for reduction of excessive height, surgery is mostly performed at a leg which has to be considered as “non-pathological”. However, the results of the existing studies [5, 6] should be interpreted with caution since, until recently, no validated reference values were available to reliably assess alterations in knee joint morphology and to determine whether they are outside of physiological ranges. The present study aims to evaluate changes in knee joint morphology after tED with two different devices and correlate the findings to age-specific radiographic reference values of the central knee anatomy [7], as well as to assess secondary deformities of the coronal plane.

## Methods

### Patients

**Fig. 1:**
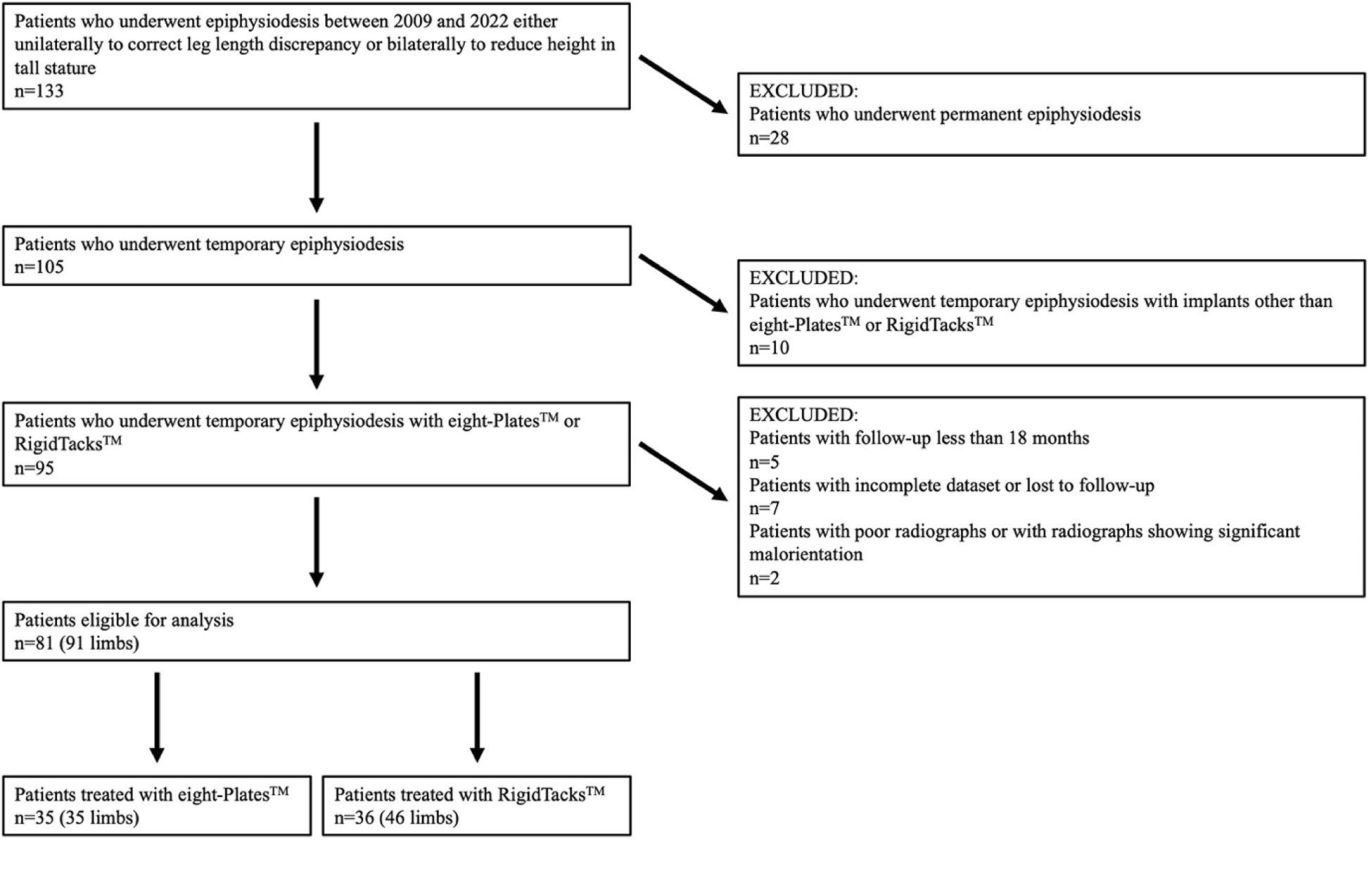
STROBE diagram depicting the inclusion and exclusion criteria of the study

Radiographs of 133 patients who underwent tED between 2009 and 2022 were retrospectively analysed. Patient demographics were retrieved from electronic patient records and are given in Table 1. tED was either conducted using dual eight-Plates^TM^ (EP; Orthofix, Lewisville, TX, USA) or RigidTacks^TM^ (RT; Merete, Berlin, Germany). Study findings are reported according to the Strengthening the Reporting of Observational Studies in Epidemiology guidelines (Fig. 1) [8]. Eighty-one patients (31 females and 50 males) who underwent tED fulfilled the inclusion criteria and were available for analysis. Ten of 81 patients (12%) received bilateral tED with RT “around the knee” for the reduction of excessive predicted final height in tall stature. In 71 of 81 patients (88%) tED was performed unilaterally for correction of LLD. In 35 of 71 patients (49%) tED was undertaken with EP, and in 36 (51%) with RT. tED was done bisegmentally in the distal femur and proximal tibia in 45 of 71 patients (63%), solely in the distal femur in 17 of 71 patients (24%), and solely in the proximal tibia in nine of 71 patients (13%) (Table 1).

**Table 1:** Patient demographics

	**eight-Plates**^**TM**^	**RigidTacks**^**TM**^
**Patients**	35 (43%)	46 (57%)
**Limbs**	35 (38%)	56 (62%)
**Sex** (female/male)	12/23	19/27
**Site** (tibial/femoral/tibial and femoral)	6/1/28	3/16/37
**Side** (left/right/bilateral)	15/20/0	14/22/10
**Age at surgery** (years)	12.3 (1.9)	12.5 (1.4)

Data is presented in absolute and relative numbers and as mean with standard deviation

The underlying pathologies of LLD are given in Table 2.

**Table 2:** Aetiologies of leg length discrepancies

**Aetiology**	71 (100%)
Idiopathic	23 (32%)
Hemihypertrophy	18 (25%)
Posttraumatic	9 (13%)
Lateral longitudinal deficiency	5 (7%)
Clubfoot	4 (6%)
Post tumour	3 (4%)
Legg-Calvé-Perthes disease	3 (4%)
Neuromuscular	2 (3%)
Postinfectious	2 (3%)
Neurofibromatosis Type I	1 (1%)
Silver-Russel syndrome	1 (1%)

Data is presented in absolute and relative numbers

The mean age at the time of operation was 12.5 years (standard deviation (SD) 1.6), with a mean follow-up of 3.3 years (SD 1.4). The mean treatment duration, defined as the interval from device implantation to removal, was 2.7 years (SD 1.1).

### Radiological parameters

**Fig. 2:**
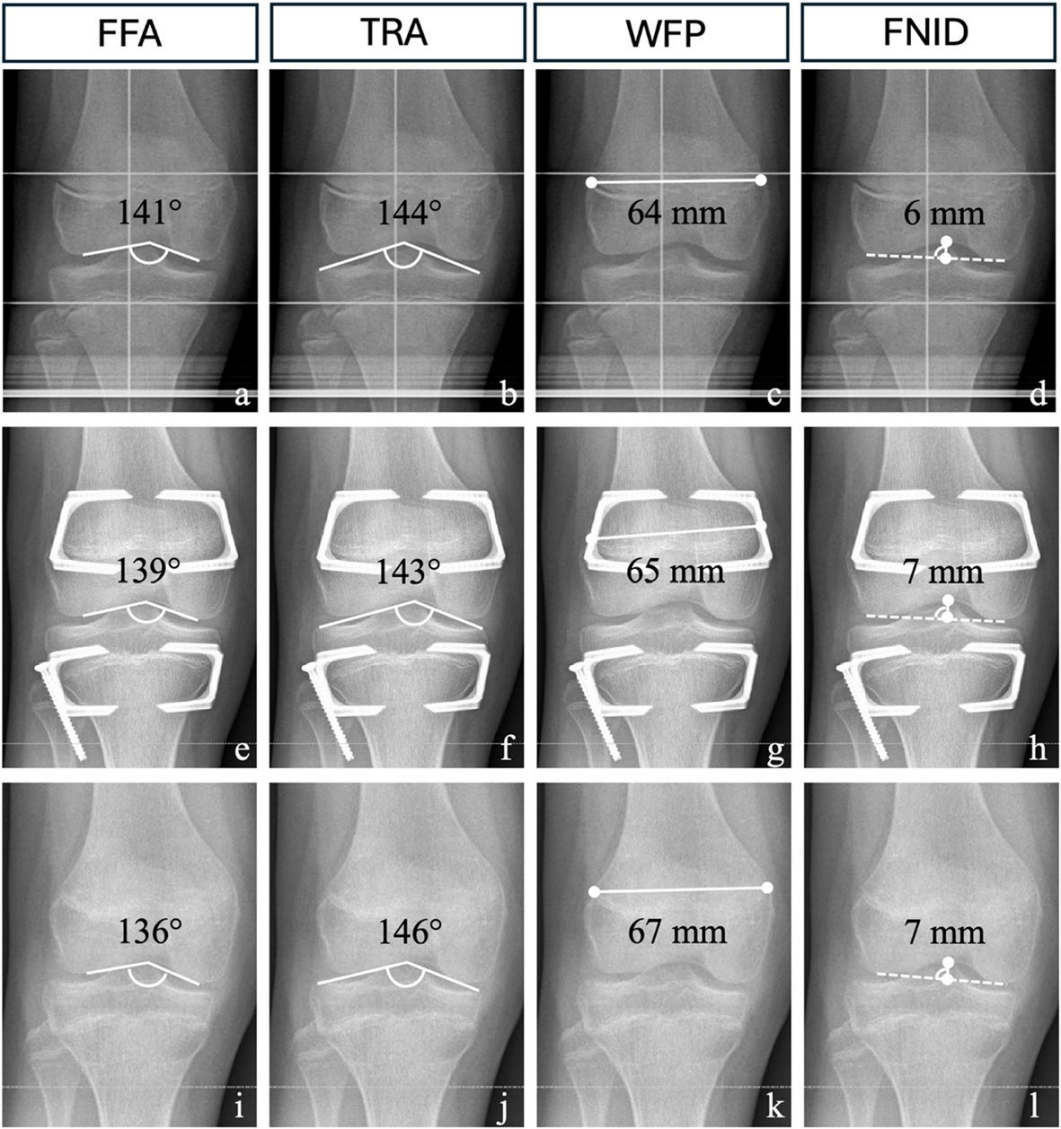
Intra-articular parameters before and after temporary epiphysiodesis. **a-d** Anteroposterior (a.p.) radiograph of the right knee joint of a 12-year-old girl with predicted idiopathic leg length discrepancy (LLD) of 2.8 cm. **e-h** a.p. radiograph of the right knee after 18 months of treatment by temporary epiphysiodesis around the knee using RigidTacks^TM^ (Merete, Berlin, Germany). **i-l** a.p. radiograph of the right knee 7 months after implant removal at last follow-up. **a** – Femoral floor angle (FFA). **b** Tibial roof angle (TRA). **c** Width at femoral physis (WFP). **d** Femoral notch-intercondylar distance (FNID). **e** FFA. **f** TRA. **g** WFP. **h** FNID. **i** FFA. **j** TRA. **k** WFP. **l** FNID

Radiological assessment was conducted preoperatively, prior to implant retrieval, and at last follow-up (Fig. 2). Implant retrieval was performed if skeletal maturation was observed, which was radiologically defined as bilateral physeal closure at the distal femur and proximal tibia, or if leg length equalisation was achieved.

Radiographic measurements were performed on calibrated anteroposterior (a.p.) long standing radiographs. The following articular morphology measurements were determined as previously reported: tibial roof angle [6] (TRA) to assess the tibial articular surface; femoral notch-intercondylar distance [9] (FNID), femoral floor angle [5] (FFA), and width at femoral physis [7] (WFP) to quantify articular changes of the distal femur. These parameters were correlated with recently established age-specific reference values [7].

To evaluate coronal alignment, the established parameters mechanical axis deviation (MAD), mechanical lateral distal femoral angle (mLDFA), medial proximal tibial angle (MPTA), and joint line convergence angle (JLCA) were analysed [10].

The software utilised for examining these parameters included the Picture Archiving and Communication System (PACS; IMPAX 6.5, AGFA HealthCare N.V., Mortsel, Belgium) and the post-processing software TraumaCad (Brainlab, Munich, Germany).

All measurements were conducted by a single observer, as no bias in intra- or interobserver reliability has been demonstrated [7, 11, 12].

### Implants and surgical technique

The respective implants were inserted minimally invasively, both medially and laterally, at the distal femur and/or proximal tibia. EP and RT were implanted and removed according to the developer’s and manufacturer’s guidelines, preserving the periosteum [4, 13].

Following implantation and removal, immediate full weight-bearing as tolerated was permitted. Routine clinical and radiographic examinations with a.p. long standing radiographs were conducted every six months.

### Statistical analysis

Statistical analyses were performed using the SPSS Statistics 29.0 (IBM SPSS, Armonk, NY, USA). For descriptive analysis, patient characteristics were presented in absolute and relative numbers for categorical variables or as mean and SD for normally distributed continuous variables and as median and interquartile range presented as 25^th^–75^th^ percentile for non-normally distributed continuous variables.

Comparisons between both implant types, EP and RT, and the outcome variables, specifically the differences [after vs. before surgery] and [last follow-up vs. before surgery] values for FFA, TRA, WFP, FNID, MAD, MPTA, mLDFA, and JLCA were calculated using unpaired t test or Mann-Whitney U test. Additionally, for all outcome differences, regression models were performed to examine if, alongside the implant type, different covariates – such as gender, bone, chronological age at operation, treatment duration, achieved growth reduction, and aetiology – were influencing variables on the outcomes. Results were presented in 95% confidence intervals (95% CI). Linear mixed models were fitted to account for the dependence in the data due to the different time points from one patient. This was done with a random effect for the patient, using a working correlation matrix with an unstructured format.

As this was an investigative study, no adjustments for multiple testing were made. The statistical tests were performed for illustrative purposes only. P‑values were reported for descriptive reasons only but were considered significant if they were lower than 0.05. All p-values should be interpreted with caution and in conjunction with effect estimates and confidence limits.

## Results

Fifty-one of 81 patients (63%) had achieved skeletal maturity at the time of last follow-up. LLD before plate insertion and at the end of the correction period, as well as the achieved growth reduction in tall stature patients, are presented in Table 3.

**Table 3:** Treatment parameters

	**eight-Plates**^**TM**^	**RigidTacks**^**TM**^
**Treatment duration** (years)	2.6 (2.0–3.2)	2.6 (0.9)
**Follow-up** (years)	3.6 (1.7)	3.1 (1.1)
**LLD before treatment** (cm)	3.0 (2.2–3.5)	2.7 (1.9–2.6)
**LLD after treatment** (cm)	1.1 (1.4)	0.8 (0–1.1)
**LLD difference after vs. before treatment** (cm)	1.8 (1.3)	1.5 (0.9–2.5)
**Achieved growth reduction in tall stature patients** (cm)	-	9.6 (7.4)
**Secondary deformities** (valgus deformity/varus deformity)	3/1	0/7

Data is presented in absolute numbers, as mean with standard deviation and as median with interquartile range presented as 25^th^–75^th^ percentile. LLD: leg length discrepancy

### Radiological parameters

Prior to implant removal, statistically significant changes were observed for the FNID, WFP, and MAD within the EP group. In contrast, the RT group demonstrated significant changes solely for the FNID. Specifically, the EP group recorded a mean increase in the FNID of 1.0 mm (SD 1.5), while the RT group exhibited a mean increase of 0.6 mm (SD 1.3). Furthermore, the WFP increased by a mean of 3.5 mm (SD 5.2) in the EP group, whereas the RT group showed a mean increase of 0.9 mm (SD 3.1). The MAD shifted by a mean of +3.9 mm (SD 7.1) in the EP group, in contrast to a mean change of only +1.9 mm (SD 8.8) in the RT group. Notably, no significant changes were detected in the other assessed parameters (Tables 4 and 5).

**Table 4:** Intra-articular parameters before and after temporary epiphysiodesis

	**eight-Plates**^**TM**^	**RigidTacks**^**TM**^
**FFA before treatment** (°)	135 (10.2)	132 (8.4)
**FFA after treatment** (°)	136 (8.0)	131 (9.2)
**FFA at last follow-up** (°)	135 (8.4)	130 (8.7)
**FFA difference after vs. before treatment** (°)	0.6 (−5.0–3.8)	0.7 (−5.2–3.8)
**p-value**	0.776	0.759
**TRA before treatment** (°)	143 (4.7)	145 (4.0)
**TRA after treatment** (°)	142 (5.3)	146 (4.1)
**TRA at last follow-up** (°)	142 (5.2)	146 (4.0)
**TRA difference after vs. before treatment** (°)	−0.7 (−1.7–3.1)	0.5 (−2.2–1.3)
**p-value**	0.571	0.601
**WFP before treatment** (°)	70.8 (6.3)	75.3 (7.3)
**WFP after treatment** (°)	74.3 (6.4)	76.3 (6.6)
**WFP at last follow-up** (°)	75.5 (6.2)	75.5 (7.7)
**WFP difference after vs. before treatment** (°)	3.5 (−6.5–−0.5)	0.9 (−3.9–2.1)
**p-value**	**0.025**	0.559
**FNID before treatment** (°)	6.9 (1.3)	7.6 (1.3)
**FNID after treatment** (°)	7.8 (1.6)	8.5 (1.7)
**FNID at last follow-up** (°)	7.9 (1.6)	8.5 (1.7)
**FNID difference after vs. before treatment** (°)	1.0 (−1.7–−0.3)	0.6 (−1.3–−0.2)
**p-value**	**0.008**	**0.043**

Data is presented as mean with standard deviation and as mean with 95% confidence interval

*FFA* femoral floor angle, *TRA* tibial roof angle, *WFP* width at femoral physis, *FNID* femoral notch-intercondylar distance

**Table 5:** Frontal plane alignment before and after temporary epiphysiodesis

	**eight-Plates**^**TM**^	**RigidTacks**^**TM**^
**MAD before treatment** (mm)	5.7 (3.9)	6.4 (4.8)
**MAD after treatment** (mm)	9.6 (6.1)	8.3 (7.9)
**MAD at last follow-up** (mm)	7.9 (4.9)	7.0 (5.6)
**MAD difference after vs. before treatment** (mm)	3.9 (−6.3–−1.4)	1.9 (−4.5–0.7)
**p-value**	**0.002**	0.154
**mLDFA before treatment** (°)	87 (2.1)	87 (2.0)
**mLDFA after treatment** (°)	87 (2.8)	88 (2.7)
**mLDFA at last follow-up** (°)	87 (2.3)	88 (2.4)
**mLDFA difference after vs. before treatment** (°)	−0.2 (−1.0–1.3)	0.1 (−1.1–1.0)
**p-value**	0.772	0.920
**MPTA before treatment** (°)	88 (2.2)	89 (2.4)
**MPTA after treatment** (°)	87 (2.9)	88 (2.9)
**MPTA at last follow-up** (°)	88 (2.7)	88 (2.6)
**MPTA difference after vs. before treatment** (°)	−0.7 (−0.6–1.9)	−0.4 (−0.7–1.5)
**p-value**	0.287	0.496
**JLCA before treatment** (°)	1.1 (0.9)	1.1 (0.9)
**JLCA after treatment** (°)	1.4 (1.1)	1.3 (0.9)
**JLCA at last follow-up** (°)	1.3 (1.1)	1.2 (0.9)
**JLCA difference after vs. before treatment** (°)	0.3 (−0.8–0.1)	0.1 (−0.5–0.3)
**p-value**	0.166	0.596

Data is presented as mean with standard deviation and as mean with 95% confidence interval

*MAD* mechanical axis deviation, *mLDFA* mechanical lateral distal femoral angle, *MPTA* medial proximal tibial angle, *JLCA* joint line convergence angle

When compared to reference values for untreated healthy children and adolescents, the parameters TRA, WFP, and FNID exhibited average values within the expected range for the entire cohort as well as in the subgroup analysis, both prior to implantation and before device removal. Although the mean FFA remained below the average, it was still within one SD, indicating a generally acceptable range. In nine patients who underwent isolated tED of the proximal tibia, two exhibited morphological changes in the distal femur (FFA). Relevant changes were characterised by a divergence of more than one SD from the mean.

The dataset was further segmented into age groups for EP and RT patients to adjust for age-related variations and facilitate comparisons with the reference values. Consistent with the overall analysis, the parameters TRA, WFP, and FNID continued to fall within the defined physiological range. FFA remained below average across both patient groups and all age categories, further corroborating the findings. All results are detailed in Tables 6 and 7.

**Table 6:** Comparison of intra-articular parameters with age-specific reference values [7]

**FFA before treatment**			
**Age group** (years)	**Reference value** (°)	**eight-Plates**^**TM**^ (°)	**RigidTacks**^**TM**^ (°)
8–10	144 (143–146)	141 (131–153) n=6	133 (123–150) n=11
11–12	143 (142–144)	137 (115–150) n=14	131 (107–146) n=25
13–14	141 (140–142)	132 (109–148) n=11	133 (120–145) n=20
15–16	140 (138–142)	136 (133–139) n=3	-
**FFA after treatment**			
**Age group** (years)	**Reference value** (°)	**eight-Plates**^**TM**^ (°)	**RigidTacks**^**TM**^ (°)
8–10	144 (143–146)	145 (143–147) n=2	-
11–12	143 (142–144)	124 n=1	142 n=2
13–14	141 (140–142)	138 (129–149) n=9	128 (111–142) n=18
15–16	140 (138–142)	134 (121–156) n=19	130 (108–145) n=22
**TRA before treatment**			
**Age group** (years)	**Reference value** (°)	**eight-Plates**^**TM**^ (°)	**RigidTacks**^**TM**^ (°)
8–10	140 (139–141)	140 (136–143) n=6	143 (137–151) n=11
11–12	144 (143–145)	142 (138–150) n=14	146 (140–153) n=25
13–14	145 (145–146)	145 (138–154) n=11	146 (140–157) n=20
15–16	147 (145–148)	144 (141–150) n=3	-
**TRA after treatment**			
**Age group** (years)	**Reference value** (°)	**eight-Plates**^**TM**^ (°)	**RigidTacks**^**TM**^ (°)
8–10	140 (139–141)	133 (132–133) n=2	-
11–12	144 (143–145)	143 n=1	144 (141–146) n=2
13–14	145 (145–146)	140 (135–149) n=9	145 (137–157) n=18
15–16	147 (145–148)	144 (135–152) n=19	146 (140–153) n=22

Data is presented in absolute numbers and as mean with 95% confidence interval

*FFA* femoral floor angle, *TRA* tibial roof angle. One patient in the eight-Plate™ group was younger than eight years at the time of surgery (7.9 years) and therefore was not included in the “8–10 years” age category. Additionally, four patients in the RigidTack^TM^ group older than 18 years were not included in the “after treatment” categories, as they did not fit within the “17–18 years” age category

**Table 7:** Comparison of width at femoral physis and mechanical axis with reference values [7, 10]

**WFP before treatment**			
**Age group** (years)	**Reference value** (mm)	**eight-Plates**^**TM**^ (mm)	**RigidTacks**^**TM**^ (mm)
8–10	69 (68–70)	66.6 (57.1–70.6) n=6	69.2 (63.9–75.2) n=11
11–12	72 (71–73)	69.8 (61.8–77.2) n=14	75.3 (63.2–88) n=25
13–14	76 (74–77)	74.8 (64.8–87) n=11	78.7 (63.8–92.6) n=20
15–16	76 (74–77)	74.2 (73.1–75.4) n=3	-
**WFP after treatment**			
**Age group** (years)	**Reference value** (mm)	**eight-Plates**^**TM**^ (mm)	**RigidTacks**^**TM**^ (mm)
8–10	69 (68–70)	69.6 (64.1–75) n=2	-
11–12	72 (71–73)	66.9 n=1	70.9 (69.1–72.6) n=2
13–14	76 (74–77)	77.1 (68.4–86.7) n=9	75.2 (64.2–89.7) n=18
15–16	76 (74–77)	74.2 (61.6–84.4) n=19	76.5 (60.7–88.3) n=22
**MAD before treatment**			
**Age group** (years)	**Reference value** (mm)	**eight-Plates**^**TM**^ (mm)	**RigidTacks**^**TM**^ (mm)
8–10	7 (6.5–7)	6.0 (4.1–7.5) n=6	6.4 (5.1–7.7) n=11
11–12	8 (7–8.8)	6.4 (4.3–8.1) n=14	7.9 (6.1–10.3) n=25
13–14	9 (7.7–9.7)	7.8 (6.1–10.1) n=11	8.0 (5.8–11) n=20
15–16	8 (7.4–8.3)	7.2 (6.6–8.4) n=3	-
**MAD after treatment**			
**Age group** (years)	**Reference value** (mm)	**eight-Plates**^**TM**^ (mm)	**RigidTacks**^**TM**^ (mm)
8–10	7 (6.5–7)	6.8 (5.8–7.7) n=2	-
11–12	8 (7–8.8)	9.5 n=1	6.6 (6.1–7.1) n=2
13–14	9 (7.7–9.7)	7.6 (4.9–9.8) n=9	8.4 (6.3–13.3) n=18
15–16	8 (7.4–8.3)	8.0 (2.8–10.7) n=19	8.7 (6.0–11.5) n=22

Data is presented in absolute numbers and as mean with 95% confidence interval

*WFP*: width at femoral physis, MAD: mechanical axis deviation. One patient in the eight-Plate™ group was younger than eight years at the time of surgery (7.9 years) and therefore was not included in the “8–10 years” age category. Additionally, four patients in the RigidTack^TM^ group older than 18 years were not included in the “after treatment” categories, as they did not fit within the “17–18 years” age category

A range of potential confounders has been identified throughout the study. The dataset was scrutinised for the influence of various factors, including patient age at the time of operation, gender, duration of treatment, achieved growth correction, type of bones treated, underlying aetiology, and the implants used. Specifically, regarding the FFA, the choice of implants (regression model, p=0.026, estimate +5.231 EP vs. RT, 95% CI 0.6–9.8), and the patient's age (regression model, p=0.02, estimate 1.643, 95% CI 0.3–3.0) were acknowledged as confounders at the time of device removal. Notably, no significant confounders were detected prior to implantation. Throughout the procedure, treatment duration had a significant impact on both the FFA (regression model, p=0.046, estimate −1.483, 95% CI −2.9–−0.3) and the TRA (regression model, p=0.012, estimate 1.122, 95% CI 0.3–2.0). In contrast, the patient's age at the time of surgery did not serve as a confounding variable (FFA: regression model, p=0.593, estimate 0.299, 95% CI −0.8–1.4; TRA: regression model, p=0.388, estimate 0.286, 95% CI −0.4–0.9). Interestingly, EP patients displayed a lower TRA compared to RT patients before implantation (regression model, p=0.05, estimate −2.307 EP vs. RT, 95% CI −4.6–0.002). However, this difference was not statistically significant until device removal, at which point it achieved significance (regression model, p=0.013, estimate −3.234 EP vs. RT, 95% CI −5.8–−0.7). Additionally, the site of tED (regression model, p=0.022, estimate −4.207 tibial vs. femoral and tibial, 95% CI −7.8–−0.6) was found to influence the TRA at the time of device removal.

The classification of different implant types was identified as a confounding factor affecting WFP (regression model, p=0.04, estimate −3.799 EP vs. RT, 95% CI −7.4–−0.2) prior to implantation. During the procedure, the implant type (regression model, p=0.035, estimate 2.061 EP vs. RT, 95% CI 0.1–4.0), gender (regression model, p=0.007, estimate −2.232 female vs. male, 95% CI −3.8–−0.6), age at surgery (regression model, p<0.001, estimate −1.601, 95% CI −2.2–−1.0), and the site of tED (regression model, p=0.036, estimate 2.807 tibial vs. femoral and tibial, 95% CI 0.2–5.4) were found to influence WFP. During the procedure, gender (regression model, p=0.042, estimate −0.671 female vs. male, 95% CI −1.3–−0.03) and the achieved growth reduction (regression model, p<0.001, estimate 0.144, 95% CI 0.06–0.2) had an impact on FNID. Finally, age (regression model, p=0.014, estimate −1.738, 95% CI −3.1–−0.4) and the achieved growth reduction (regression model, p=0.002, estimate −0.779, 95% CI −1.3–−0.3) were identified as confounders influencing MAD during the procedure.

### Secondary deformities

**Fig. 3:**
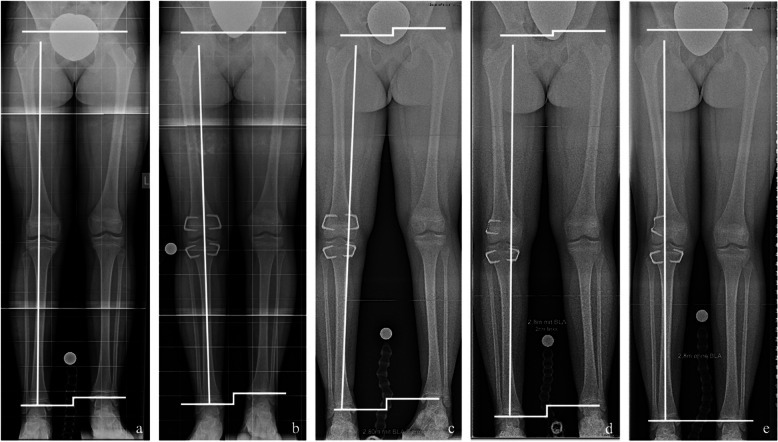
Secondary coronal angular deformity after temporary epiphysiodesis. **a** Anteroposterior long standing radiograph of a 10-year-old girl with predicted idiopathic leg length discrepancy (LLD) of 3 cm. **b** After temporary epiphysiodesis around the right knee using RigidTacks^TM^ (Merete, Berlin, Germany) showing neutral coronal alignment of the treated right leg. **c** After 16 months of treatment showing significant secondary femoral varus deformity of the right leg. **d** After conversion to a distal femoral hemiepiphysiodesis through implant removal on the concave side of the deformity and change to a flexible staple (FlexTack^TM^, Merete, Berlin, Germany). **e** After another 6 months of treatment showing restoration of the neutral coronal alignment of the right leg and complete LLD equalisation

Revision surgery due to secondary coronal angular deformity was required in four of 35 patients (11%) treated with EP and in seven of 46 patients (15%) treated with RT. All seven patients treated with RT presented varus malalignment, while this was the case in three patients in the EP group, with one patient developing valgus deformity. Limb realignment was achieved in all patients by converting to a hemiepiphysiodesis through partial implant removal on the concave side of the deformity (Fig. 3). Five of eleven secondary deformities (45%) were observed in patients with congenital aetiologies.

No clinical signs of sagittal plane deformities such as hyperextension of the knee were observed. Therefore, we refrained from taking additional radiographs in the lateral view.

## Discussion

While the risk of secondary coronal malalignment has been thoroughly investigated [1, 2, 14–16], potential intra-articular deformities after tED have only recently come into focus [5, 6]. It has been suspected that implant-mediated tED at the medial and lateral aspect of the growth plate may lead to changes in central knee joint morphology due to tethering at the physeal periphery, resulting in sustained central physeal growth [5]. Sinha et al. were among the first to report on epiphyseal deformities of the proximal tibia after tED with dual tension-band plating in patients with LLD. They found a decreased TRA and termed their observation of central protrusion and peripheral flattening the “volcano deformity” [6]. A relevant decrease of the TRA after tED to correct LLD has also been found by Starobrat et al. [17] and Tolk et al. [5], while Jain et al. did not find significant changes of the TRA [18]. Tolk et al. also investigated the effect of tED on the morphology of the distal femur. In line with our findings, they only observed a relevant increase of the FNID. However, it should be noted that the difference between both legs was only 0.5 mm. Consequently, the authors did not interpret this as a significant change in intra-articular morphology of the distal femur [5]. Moreover, with sustained central physeal growth and peripheral physeal tethering, a decrease in the FNID rather than an increase should be expected. Furthermore, it remains unclear whether monosegmental tED may influence the growth of the corresponding untreated segment. In the present study, morphological changes in the distal femur were observed in two of nine patients who underwent isolated tED of the proximal tibia. However, in accordance with the observation by Jain et al. [18] there were no significant changes in the TRA, regardless which segment had been treated.

Thus far, it is not known whether the morphological changes of the proximal tibia after tED observed by several study groups [5, 6, 17] are a merely radiological phenomenon, or if they imply any clinical consequences, such as knee pain or early-onset osteoarthritis [5]. Additionally, it should be questioned whether the reported changes in the TRA should be considered pathological at all. Comparing their findings to established reference values, the reported TRA appears to remain within one SD of the age-specific reference values in all cases [5, 7, 17]. In the present study, relevant changes of the TRA were not observed at all, and the relevant changes of the FNID and WFP that were found were within one SD of the age-specific reference values [7]. These findings suggest that the morphological changes of the distal femoral epiphysis after tED lie within physiological ranges and are most likely without pathological implications. Moreover, the observed changes of the WFP may be merely attributed to continued bone growth and is thus observed after longer treatment or follow-up time. However, treatment duration of tED may indeed contribute to changes in epiphyseal morphology. Starobrat et al. found the greatest change in the TRA after 42 months of tibial tED [17]. It remains unknown whether the observed radiological changes may be reversible if implants are removed before skeletal maturity. It may be possible that a growth acceleration at the medial and lateral physis, following the release of the tethering [1], may lead to a “normalisation” of the bony morphology. Accelerated growth is frequently observed after temporary hemiepiphysiodesis for treatment of angular deformities, which may result in rebound deformity if there is sustained longitudinal growth [19]. Conversely, Tolk et al. observed a slightly slower growth rate after plate removal at the distal femur, with no changes in the growth rate of the proximal tibia [5]. This effect may differ after tED with rigid staples, which cause rigid physeal compression and potentially complete growth arrest, as opposed to the growth deceleration induced by dual tension-band plating [4, 20, 21]. Consequently, there may also be a reduced risk for the development of intra-articular deformities in tED using rigid staples [4, 21]. However, in the present study, even though there were more relevant changes following tED with tension-band plates compared to rigid staples, all changes remained within physiological ranges. This suggests that there are no significant implant-related differences in regard to their effect on joint morphology. This conclusion extends to secondary deformities in the coronal plane, as the rates of coronal plane deformities requiring revision surgery were similar in both implant groups. Coronal plane deformities are a well-known complication of tED [14–16]. Gorman et al. reported that half of all patients who underwent tED using rigid staples exhibited a relevant shift in the mechanical axis of >10 mm, with 89% demonstrating varus malalignment [14]. In the present study, 73% of all secondary deformities were identified as varus deformities. Gorman et al. also noted that tED at the proximal tibia, as well as combined femoral and tibial tED, is associated with a higher risk of coronal plane deformities compared to isolated femoral tED. The authors concluded that distal femoral tED may be less vulnerable to secondary coronal malalignment [14]. Erdal et al. postulated that the risk of angular deformities may be greater with tED performed on pathological physes [15]. In our patient cohort, five out of eleven secondary coronal deformities were observed in patients with congenital aetiologies. However, it should be noted that, in most cases the leg treated with tED for LLD correction generally does not present with underlying pathologies, in particular in regard to physeal growth abnormalities. Given that permanent epiphysiodesis techniques are associated with a lower risk of coronal plane deformities compared to tED [3, 4], we postulate that the latter should be applied with caution, particularly in highly elective indications, such as the treatment of tall stature.

### Limitations

This study has several limitations. Notably, its retrospective design and reliance on radiographs obtained in a single plane are significant constraints. Since lateral views of the joint are not typically acquired during follow-up assessments, our ability to evaluate secondary deformities in the sagittal plane is limited. However, we decided against obtaining additional lateral radiographs, as the clinical examination did not reveal any sagittal plane deformities in any patient.

Additionally, we focused exclusively on the radiological morphology of the joint rather than on clinical outcomes. Comparisons with healthy untreated children should be made with caution, given the small number of patients after stratifying the groups by age ranges and implant positioning. Finally, the heterogeneity of the cohort, the underlying pathology and amount of LLD, and thus the variable treatment time may introduce bias. Furthermore, not all patients had reached skeletal maturity at the time of the last follow-up.

## Conclusions

The present study is the first to investigate secondary morphological changes in both the distal femur and the proximal tibia in tED for the treatment of LLD and tall stature, using either tension-band plates or rigid staples, while also comparing the results with established reference values. Intra-articular deformities – particularly the so-called tibial “volcano deformity” – were not observed in any of the studied cohorts. The morphological changes observed in the distal femur remained within the physiological ranges of established reference values and are likely growth-related. However, the relevant occurrence of secondary coronal plane deformities should be considered when choosing the appropriate treatment.

## Data Availability

The datasets used and/or analysed during the current study are available from the corresponding author on reasonable request.

## Abbreviations

a.p.: Anteroposterior
CI: Confidence interval
cm: Centimetre
EP: eight-Plate^TM^
et al.: Et alii
FFA: Femoral floor angle
FNID: Femoral notch-intercondylar distance
JLCA: Joint line convergence angle
mLDFA: Mechanical lateral distal femoral angle
LLD: Leg length discrepancy
M.D.: Medical doctor
MAD: Mechanical axis deviation
mm: Millimetre
MPTA: Medial proximal tibial angle
RT: RigidTack^TM^
SD: Standard deviation
tED: Temporary epiphysiodesis
TRA: Tibial roof angle
vs.: Versus
WFP: Width at femoral physis
